# Theoretical Modeling and Experimental Analysis of Single-Particle Erosion Mechanism of Optical Glass

**DOI:** 10.3390/mi12101221

**Published:** 2021-10-06

**Authors:** Zhongchen Cao, Shengqin Yan, Shipeng Li, Yang Zhang

**Affiliations:** 1 Key Laboratory of Advanced Ceramics and Machining Technology, Ministry of Education, Tianjin University, Tianjin 300072, China; ysq@tju.edu.cn (S.Y.); 14221278@bjtu.edu.cn (S.L.); summer0724zy@icloud.com (Y.Z.); 2 Key Laboratory of Mechanism Theory and Equipment Design of Ministry of Education, Tianjin University, Tianjin 300072, China

**Keywords:** single-particle erosion, smoothed particle hydrodynamics, mechanism, optical glass, subsurface damage

## Abstract

The study of the single-particle erosion mechanism is essential to understand the material removal mechanism in the non-contact polishing process and ultimately ensure the high-efficiency, non-damage, and ultra-smooth processing of optical glass. In this study, the theoretical model of smoothed particle hydrodynamics (SPH) is established to reveal the dynamic removal process of a single particle impacting the optical glass. The single-particle erosion mechanisms, which include ductile–brittle transition, crack initiation, and propagation, are discussed in detail through theoretical simulation. A series of particle impact experiments are designed to validate the correctness of the SPH model. The experimental data show good agreement with the simulation results in terms of the depth and width of the eroded craters. Thereafter, the SPH simulation is conducted by studying the effect of various impact parameters, such as impact speed, impact angle, and abrasive diameter, on the material removal process. With the gradual increase of impact velocity and particle size, the material removal mode changes from plastic removal to brittle removal. Although the large impact velocity and particle size increase the material removal rate, they lead to the occurrence of brittle removal and reduce the surface and sub-surface quality. When the impact angle is between 45° and 75°, the material removal rate is the largest, and the increase of the material removal rate does not cause damage to the subsurface layer of the material.

## 1. Introduction

Optical glass has become extensively used in aerospace, defense energy, and microelectronics due to its excellent material properties, such as high-temperature resistance, corrosion resistance, high strength, and good wear resistance [1,2]. To satisfy the stringent precision requirements of modern optical applications, the optical glass should be efficiently fabricated into ultra-smooth surface and low subsurface damage [3]. However, due to its high brittleness and low fracture toughness, brittle fracture is inevitably introduced during the processing of optical glass, which affects its ultimate performance [4,5]. Ultra-precision polishing technology such as fluid jet polishing, abrasive air jet polishing, and disc hydrodynamic polishing can be successfully used to suppress the occurrence of brittle fractures of optical glasses and obtain an ultra-smooth surface [6,7,8]. During these polishing processes, optical materials are removed by repeated impacts of abrasive particles in the polishing slurry [9,10]. Therefore, studying the single-particle erosion mechanism is necessary to improve our understanding of the material removal and damage control mechanism in the non-contact polishing process, as well as achieve the high-efficiency, damage-free, and ultra-smooth processing of optical glasses.

Many experimental investigations of particle impact tests under static and dynamic contact loads have been conducted to understand the erosion mechanism of brittle materials [11,12,13,14]. Evans et al. [15] found that the abrasive erosion mechanism of optical glass is similar to the static indentation mechanism. Lawn et al. [16] further analyzed the mechanism of crack initiation and propagation based on a static indentation experiment. Zhao et al. [17] identified three abrasive–workpiece interaction stages, namely, elastic, plastic, and brittle stages, during the abrasive erosion process. Qi et al. [18] conducted a series of microparticle impact tests and found three categories of the eroded impressions, including craters, scratches, and microdents. In their study, the influence of different impact speeds on the material removal rate was also studied. Hadavi et al. [19] analyzed the effect of impact parameters including impact speed, abrasive size, and incident angle on the brittle fracture and rebound kinematics. They found more larger abrasive fractures than smaller ones at the same impact speed. Although related scholars have conducted experimental research on the erosion mechanism of hard and brittle materials, the erosion theory cannot explain the erosion of micro- and nano-abrasive particles in ultra-precision polishing technology due to the large size of the abrasive particles used in the study. In addition, the experimental study can only be performed by observing the surface and subsurface characteristics of the hard and brittle materials after particle impact, and cannot achieve real-time monitoring of various dynamic information during the impact process, such as crack initiation and growth, stress, phase change, and temperature [18].

To observe the dynamic removal process of abrasive particle erosion, researchers have used numerical simulation methods to study the material removal mechanism of single-particle impact [20,21]. The finite element method (FEM) has been widely used to simulate the erosion removal of ductile and brittle materials [22,23]. Aquaro and Fontani [24] established a FEM simulation model for particle erosion of the ductile and brittle material to study the erosion mechanism of abrasive particles. Yand and Wang [25] developed a finite element model of the erosion by using Johnson–Cook and Johnson–Holmquist material models so as to study the effect of angle and speed on the erosion process. The FEM simulation model was effectively verified by comparing the experimental results with the simulation results [25]. However, the FEM model may result in mesh distortion of large deformation in simulating brittle materials. To effectively simulate the erosion removal process of brittle materials, smooth particle hydrodynamics (SPH), a meshless method, was proposed [26,27]. This method is particularly suitable for solving high-speed collisions and dynamic large deformation problems [28]. Du et al. [29] established a coupled FEM/SPH model to simulate the impact process of angular particles on the float glass. Crack propagation during the erosion process has been thoroughly characterized. This study shows the advantage of SPH in simulating brittle fracture behaviors of glass during the abrasive erosion process. Nishikawa et al. [30] studied the influence of various particle shapes, including cubic and spherical particles, on the erosion behavior of glass based on a coupled FEM/SPH model. The material removal impacted by spherical particles was greater than that of cubic particles. Hao et al. [31] analyzed the mechanism of crack initiation and propagation during the impact process of angular particles on float glass by establishing a coupled FEM/SPH model, and found that incident orientation plays a key role in the erosion process. Dong et al. [32,33] established the SPH constitutive relationship formula describing the plastic behavior and ductile fracture process, and used the improved SPH model to simulate the erosion process of particles with sharp corners. At present, research on the erosion removal mechanism of optical glasses is insufficient, and the influence mechanism of different impact parameters on the material removal process remains to be clarified. Therefore, this study develops the SPH model for single-particle erosion of optical glass, and conducts a theoretical study of various impact parameters, such as impact speed, impact angle, and abrasive diameter, on the material removal process. The single-particle erosion mechanisms, including ductile–brittle transition, crack initiation, and propagation, are discussed in detail through theoretical simulations. A series of particle impact experiments are designed to validate the correctness of the SPH model by comparing the depth and width of the eroded craters between the simulation results and the experimental data.

## 2. Smooth Particle Hydrodynamics (SPH) Simulation Model for Single-Particle Erosion of Optical Glass

### 2.1. Basic Theory of SPH Simulation

Due to its adaptability, meshlessness, Lagrangian, and particle properties, SPH provides great advantages in solving large deformations, free surface flows, and complex interface motions. As a relatively mature meshless numerical calculation method, SPH is extensively used in many engineering and scientific fields, including astrophysics, impact explosion, and hydrodynamics [34]. In SPH, the state of the system is expressed by a limited number of particles with a certain space and mass. The continuous function, f(u), that controls the range in which particles interact with one another is expressed as:

(1) $$f\left(u\right)=\int_{\Omega }^{}f\left(u^{\prime }\right)\delta \left(u-u^{\prime }\right)du^{\prime }$$

where $\delta \left(u-u^{\prime }\right)$ is the Dirac delta function, Ω is the integral space, and u is the vector of the particle position. Discrete numerical models cannot be constructed by the Dirac delta function, only with the support of “points” in the calculation process. Thus, the kernel function $T\left(u-u^{\prime },h\right)$ is used to replace the delta function, and the continuous function is modified as follows:

(2) $$\langle f(u)\rangle =\int_{\Omega }^{}f\left(u^{\prime }\right)T\left(u-u^{\prime },h\right)du^{\prime }$$

where h is the smoothing length that determines the influence area of the kernel function, which should meet the requirements of the normalization condition, Delta function property, and compact condition. As the spline kernel function has good accuracy for the three cases, it is universally used in SPH and can be expressed as:

(3) $$T(u-u^{\prime },h)=\frac{1}{h^{d}}\theta \left(\frac{\left\|u-u^{\prime }\right\|}{h}\right)$$

where *d* is the spatial dimension and *θ* is the spline function. The cubic B-spline function is often used as a representative of the spline function because of its good regularity [35]. This function can be expressed as:

(4) $$\theta (y)=C\times \left\{ \begin{array}{l} 1-1.5y^{2}+0.75y^{3}\;\;\;\left|y\right|\leq 1\\ 0.25(2-y^{3})\;\;\;\;\;\;\;\;\;\;\;\;\;\;\;1<\left|y\right|<2\\ 0\;\;\;\;\;\;\;\;\;\;\;\;\;\;\;\;\;\;\;\;\;\;\;\;\;\;\;\;\;\;\;\;\;\left|y\right|>2 \end{array}\right.$$

where C is the normalization constant that can be calculated by the spatial dimension (that is, y=x/h) and x is the distance between the two particles.

Through numerical calculation, the expression of the continuous integral of the function f(u) can be transformed into a cumulative summation process of SPH particles. After discretization, the approximate expression of the particle is as follows:

(5) $$\langle f(u_{a})\rangle =\overset{N}{\underset{b}{\sum }}\frac{m_{b}}{\rho_{b}}f\left(u_{b}\right)U\left(u_{a}-u_{b}^{\prime },h\right)$$

where $m_{b}$, $\rho_{b}$, and $m_{b}/\rho_{b}$ denote the mass, density, and volume of particle b, respectively. In the initial state, the smooth length, *h,* can be calculated by giving the position, velocity, and density of particle a. Particle b with a position within 2*h* is then found. The strain and strain rate between two particles can be calculated based on the spatial derivatives of position and velocity. Stress can help obtain the acceleration, and the velocity and position can be updated using Equation (6). The density and energy can be calculated in real time according to the position of the particle by Equations (7) and (8), respectively.

(6) $$\frac{dv_{a}^{\alpha }}{dt}=\overset{N}{\underset{b}{\sum }}m_{b}\left(\frac{\sigma_{a}^{\alpha \beta }}{\rho_{a}^{2}}-\frac{\sigma_{b}^{\alpha \beta }}{\rho_{b}^{2}}\right)\frac{\partial W_{ab}}{\partial u_{a}^{\beta }}$$

(7) $$\frac{d\rho_{a}}{dt}=\rho_{a}\overset{N}{\underset{b}{\sum }}\frac{m_{b}}{\rho_{b}}\left(v_{b}^{\beta }-v_{a}^{\beta }\right)\frac{\partial W_{ab}}{\partial u_{a}^{\beta }}$$

(8) $$\frac{dE_{a}}{dt}=-\frac{\sigma_{a}^{\alpha \beta }}{\rho_{a}^{2}}\overset{N}{\underset{b}{\sum }}m_{b}\left(v_{\alpha }^{b}-v_{\alpha }^{a}\right)\frac{\partial W_{ab}}{\partial u_{a}^{\beta }}$$

where α and β are space indices.

### 2.2. Johnson–Holmquist-2 (JH-2) Material Model

The JH-2 material model can accurately describe the fracture and fragmentation of brittle materials under the dynamic impact of abrasive grains [36]. This material model is mainly composed of a strength model, damage model, and state equation. The state equation is expressed by a cubic polynomial for materials with high strain rate, such as fused quartz glass. When *D* = 0, the material is undamaged, and the state equation is as follows:

(9) $$p=K_{1}\mu +K_{2}\mu^{2}+K_{3}\mu^{3}$$

where p is the hydrostatic pressure, $K_{1\;}$ is the bulk modulus, and $K_{2}$ and $K_{3}$ are the volumetric strain coefficients. Conversely, when D>0, the material is damaged, with elastic energy gradually reducing and potential energy of the material gradually increasing. The conversion from elastic energy into potential energy needs a correction, and is written as:

(10) $$\left(\Delta p_{t+\Delta t}-\Delta p_{t}\right)\mu_{t+\Delta t}+\frac{1}{2}\left(\Delta p_{t+\Delta t}^{2}-\Delta p_{t}^{3}\right)/K_{1}=\beta \Delta U$$

where β is the energy conversion coefficient and ΔU is the internal energy change.

A new strength model that uses the equivalent stress of the material as a power function of hydrostatic pressure, which is in connection with the strain rate and damage factor, is adopted in JH-2. The new strength model is expressed as:

(11) $$\sigma^{*}=\sigma_{i}^{*}-D\left(\sigma_{i}^{*}-\sigma_{f}^{*}\right)$$

where $\sigma^{*}$ is the equivalent stress, and $\sigma_{i}^{*}$ and $\sigma_{f}^{*}$ are the equivalent stresses of the undamaged and damaged materials, respectively. When D=0, $\sigma_{i}^{*}$ can be expressed as follows:

(12) $$\sigma_{i}^{*}=A{\left(p/p_{HEL}+\sigma_{t,m}/p_{HEL}\right)}^{N}\left[1+C\ln\left(\overset{*}{\varepsilon }/{\overset{*}{\varepsilon }}_{0}\right)\right]$$

where *A*, *C*, and *N* are model parameters, $\overset{*}{\varepsilon }$ is the true strain rate, ${\overset{*}{\varepsilon }}_{0}$ is the reference strain rate, $p_{HEL}$ is the pressure component of the Hugoniot elastic limit (HEL), p is the hydrostatic pressure, and $\sigma_{t,m}$ is the maximum hydrostatic pressure. The equivalent stress for complete fracture when D=1 is:

(13) $$\sigma_{f}^{*}=B{\left(p/p_{HEL}\right)}^{M}\left[1+C\ln\left(\overset{*}{\varepsilon }/{\overset{*}{\varepsilon }}_{0}\right)\right]$$

where *B* is the model parameter. The damage model of JH-2 can be expressed by:

(14) $$D=\sum_{}^{}\frac{\Delta \varepsilon_{p}}{D_{1}{\left(p/p_{HEL}+T/p_{HEL}\right)}^{D_{2}}}$$

where $\Delta \varepsilon_{p}$ is the integral of the effective plastic strain in a single cycle, and $D_{1}$ and $D_{2}$ are the damage constants of the material. We need to assess the brittle fracture of fused quartz glass. Due to the relatively low fracture toughness of fused quartz glass, the tensile strength limit is remarkably smaller than the compressive strength limit and the tensile strength is less than the yield limit of the material. Therefore, tensile strength is used to measure the material fracture.

### 2.3. SPH Model for Single-Particle Erosion of Optical Glass

**Figure 1:**
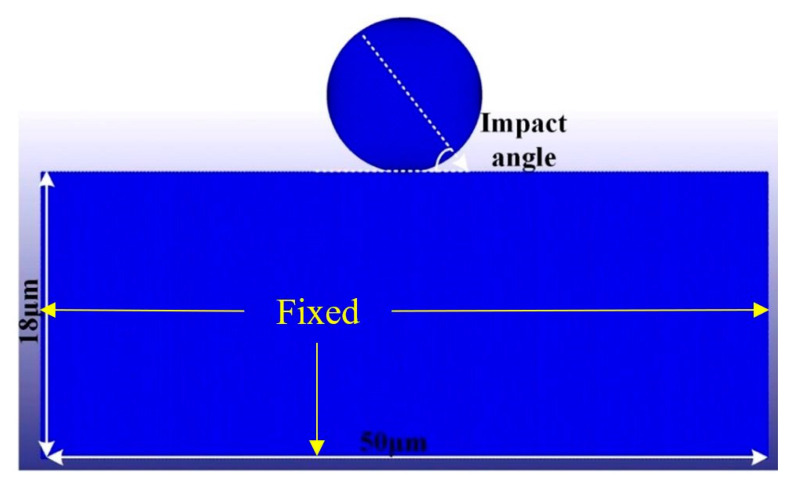
Smooth particle hydrodynamics (SPH) model of a single particle impacting the optical glass.

In the practical polishing process, the alumina particle is usually a polyhedral structure with a multiple-edge radius. To simplify the SPH simulation model of a single alumina particle impacting optical glass, we adopted spherical particles with diameters of 6, 9, and 12.8 μm. A single alumina particle is meshed using the FEM to reduce the simulation time. To balance simulation accuracy and efficiency, we designed the optical glass as a 50 × 18 × 0.3 μm cube that was divided into 60,000 SPH particles by the SPH method. The SPH geometric model of the single alumina particle impacting the optical glass is shown in Figure 1. As described in Section 2.2, the JH-2 model is applied to the optical glass material in the simulation, and relevant parameters in the JH-2 material model of the optical glass are presented in Table 1. The density, elastic modulus, and Poisson’s ratio of the single-particle alumina materials are 3.95 g/cm^3^, 350 GPa, and 0.22, respectively. The impact parameters of the simulation are shown in Table 2. The impact particles and impacted workpiece are in CONTACT-AUTOMATIC-NODES-TO-SURFACE contact mode, with the single-particle alumina being the main surface and the optical glass being the subordinate particle. BOUNDARY-SPH-SYMMETRY-PLANE is selected to fix the optical glass.

## 3. Experiments and Model Validation

### 3.1. Experimental Design

**Figure 2:**
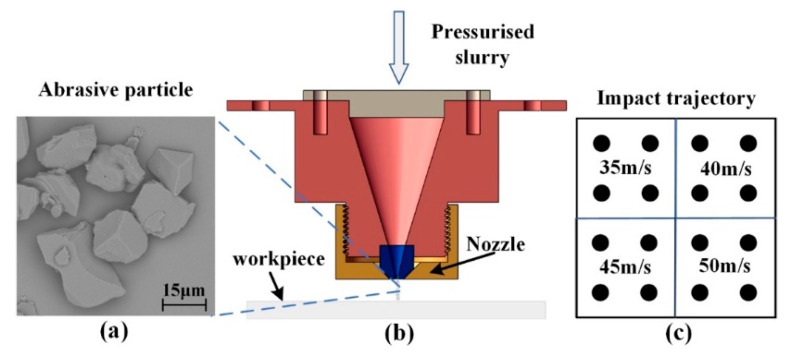
Schematic of experimental setup. (**a**) alumina particles (**b**) polishing nozzle and (**c**) impact test design.

A specific slurry supply system was used so that alumina slurry with an extremely low concentration is impacted on the surface of the optical glass in a very short time and at a specific injection pressure. Moreover, fixed-point impact on the K9 optical glass is achieved by controlling the motion control platform, and a large nozzle with a diameter of 1 mm is used to effectively identify the impact morphology of a single alumina particle. The material properties of K9 glass are density 2.5 g/cm^3^, elastic modulus 81 GPa, Vickers hardness 6.9 GPa, and fracture toughness 0.8 MPa·m^1/2^. The distance between the nozzle and optical glass is 5 mm, which is short enough for us to reasonably assume that the impact angle of a single alumina particle is equal to the incident angle. Additionally, the short impact distance and impact angle caused the velocity fluctuation of the alumina particle to become extremely small. Different impact speeds are obtained by altering the impact pressure, and calculations suggest that the corresponding impact speeds under impact pressures of 0.8, 1.0, 1.2, and 1.5 Mpa are 35, 40, 45, and 50 m/s, respectively. In order to ensure the accuracy of the experiment, the repeatability experiment was carried out by repeating each pressure condition 4 times. The distributions of impact spots under various impact pressures are shown in Figure 2.

Alumina particles (FUJIMI WA#1000) with an average diameter of 12.8 μm are used in the impact removal experiments, and the morphology of the single alumina particle is obtained through scanning electron microscopy. As shown in Figure 2a, these particles are not exactly spherical. Consequently, we can expect a certain deviation between the simulation of the SPH model, in which the alumina particles are assumed to be spherical, and the experimental data are within an allowable range. A cube workpiece (40 × 40 × 1 mm) made of optical glass was used in the experiments. A scanning electron microscope (Phenom XL, Eindhoven, The Netherlands) and a CSPM5500 scanning probe microscope (Being, Beijing, China) were used to measure the surface morphology and depth information of the craters.

### 3.2. Model Validation

**Figure 3:**
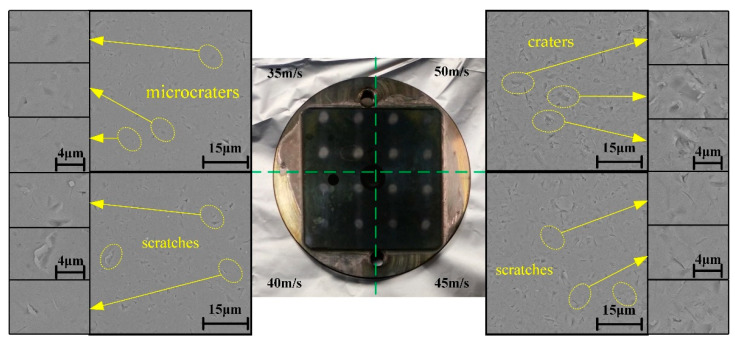
Surface morphology of impacted optical glass under different impact speeds.

Figure 3 shows the surface morphology of the optical glass material at different impact speeds. We can observe the removal characteristics (craters, scratches, and microcraters) on the surface of three types of materials, among which craters account for the largest proportion. According to the morphology, when the impact speed is 35 or 40 m/s, the surface is mainly composed of plastic craters, and the material removal mode is mainly plastic removal. However, brittle characteristics begin to form and become more dominant as the impact speed increases, indicating that brittle removal replaces plastic removal as the main removal mode. This result reveals that a lower impact speed can achieve plastic removal of the optical glass and improve the surface morphology, but the material removal rate is reduced.

**Figure 4:**
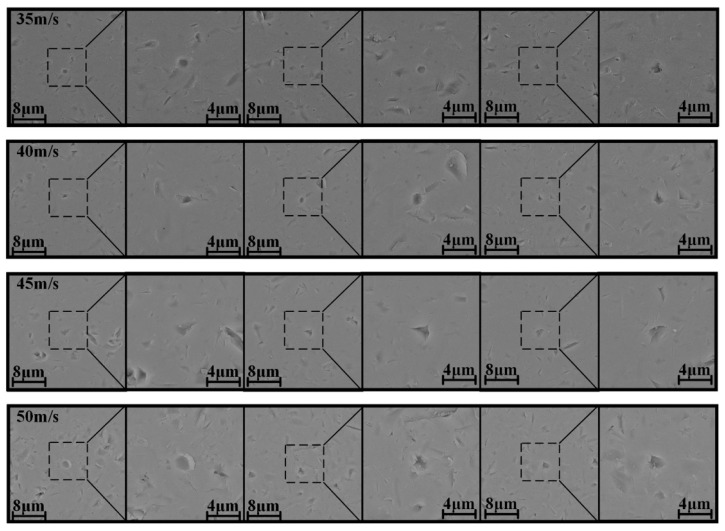
Surface morphology of a single crater under different impact speeds.

Figure 4 displays the crater morphology of the single-particle alumina impact at speeds ranging from 35 to 50 m/s. First, due to the irregularity of the shape of particles, the crater shape is not only spherical, and with the increase of the impact speed, the width of the crater also increases. At a speed no higher than 40 m/s, the removal is mainly realized by plastic extrusion, with no microcracks being generated around the craters. However, when the impact speed reaches 45 m/s or higher, microcracks begin to form around the crater. This result demonstrates that because of the increase in the impact speed, the impact energy of a single particle is greater than the critical energy of the plastic–brittle transition, thereby resulting in the appearance of microcracks. Therefore, by analyzing the crater morphology, we can conclude that the increase of the impact speed leads to the increase of the width of the craters and the formation of transverse cracks on the crater, thereby causing material breakage and affecting the surface quality of the material.

**Figure 5:**
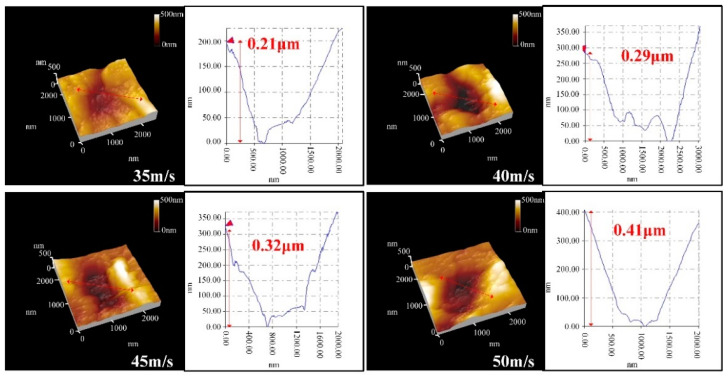
Depth information of a single crater under various impact speeds.

Figure 5 illustrates the depth information of the craters at various impact speeds. The irregularities of the shape of the impact particles cause the section of the crater to become a non-standard partial circle. With impact speed increasing from 35 to 50 m/s, we can observe that the impact depth of a single crater ranges from 0.21 to 0.41 μm. The reason is that as the impact speed increases, the impact velocity of a single particle also increases, and consequently, the increased impact kinetic energy of that particle leads to an increase in the impact depth. Thus, the increase in impact speed can increase the depth of the material removal and enhance the material removal rate.

**Figure 6:**
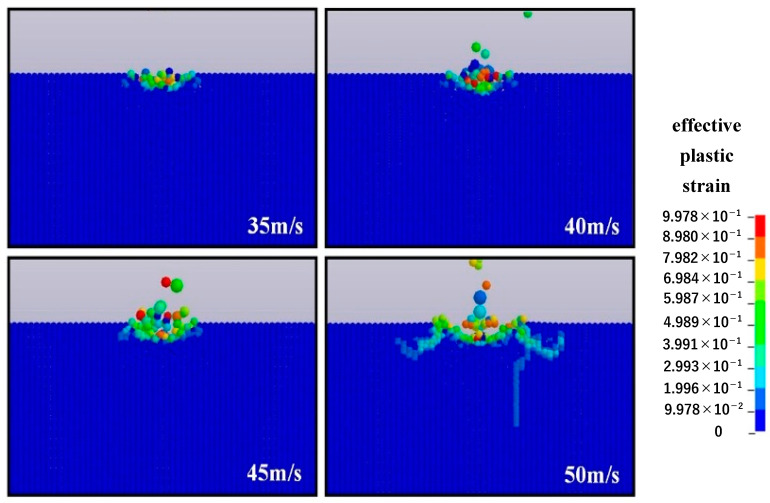
Surface morphology of impacted optical glass under different impact speeds.

Figure 6 demonstrates the SPH simulation of impact under different impact speeds, with the particle diameter being 12.8 μm and the impact angle being 90°. When the surface is impacted at the impact speed of 30 or 40 m/s, the material removal process depends on the plastic deformation of the material surface caused by extrusion between the impact particles and the surface. Nevertheless, when the impact speed reaches 45 m/s, short transverse cracks can be found, revealing that brittle removal begins to occur at this point. Moreover, as the impact velocity continues to increase, obvious brittle removal occurs: the length of the transverse cracks increases and extends to the surface of the material and radial cracks form on the subsurface. Accordingly, we can reasonably conclude that with the increase of impact speed, the material removal mode changes from plastic to brittle removal. In addition, the critical impact speed of the conversion of the removal mode is approximately 40 m/s, which is basically consistent with the experimental results.

**Figure 7:**
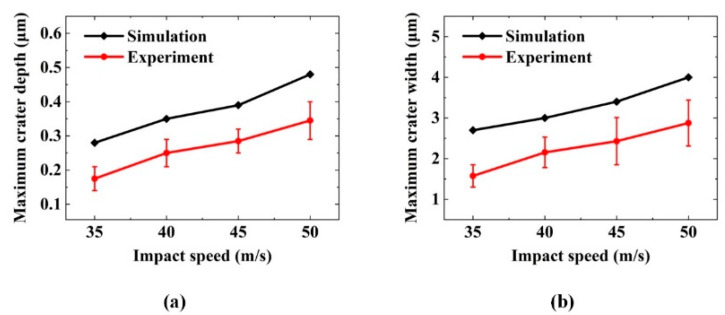
Comparison of experimental results and simulation: (**a**) change in maximum depth of crater and (**b**) change in maximum width of crater.

Figure 7 presents a comparison between the SPH simulation and the experiment of the single-particle alumina impacting the optical glass with regard to the depth and width of the removal craters. As the impact speed increases, the maximum crater depth of the simulation ranges from 0.28 to 0.48 μm, while the experimental results are between 0.21 and 0.41 μm, which show good agreement with the simulation in terms of trends and values. For the maximum crater width, the experiment is basically in accordance with the simulation: with the impact speed increasing from 35 to 50 m/s, and the width increasing from 1.6 to 2.9 μm in the experiment and from 2.7 to 4.0 μm in the simulation. Owing to the instability of nozzle pressure and the irregularity of alumina particles in the removal experiment, the data cannot fully fit the simulation but are within the allowable error range. Thus, the correctness of the SPH simulation model is verified by comparing the experimental data with the simulation.

## 4. Simulation Results and Discussion

### 4.1. Material Removal Mechanism

**Figure 8:**
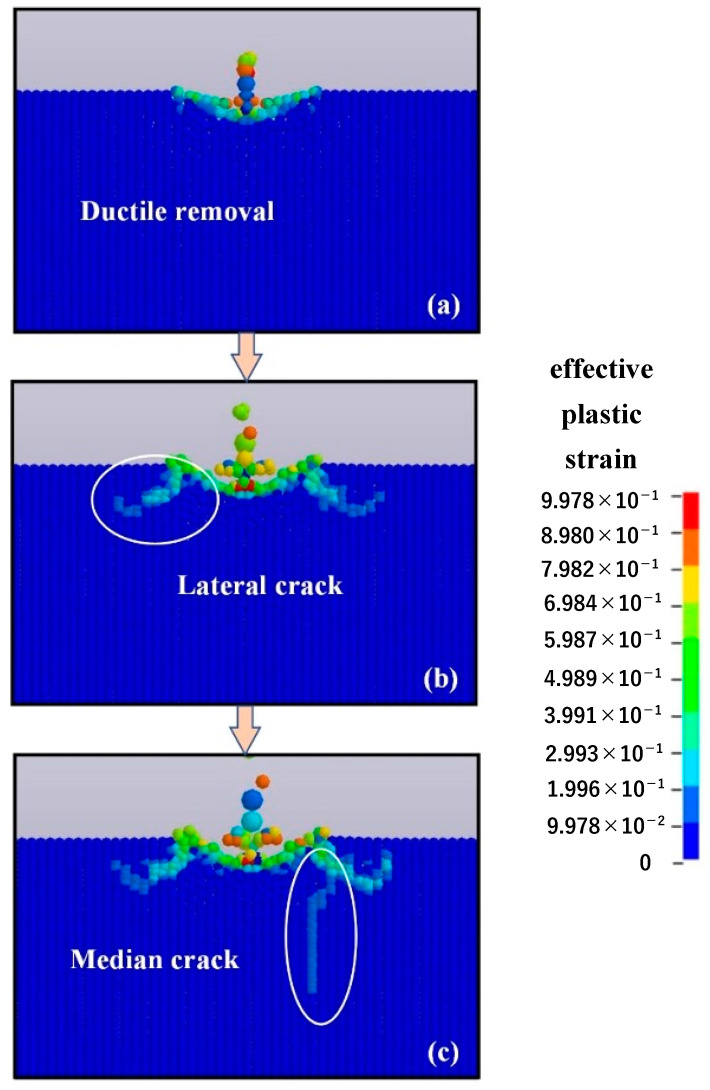
Erosion removal process of optical glass: (**a**) ductile removal, (**b**) lateral crack, and (**c**) radial crack.

Figure 8 illustrates the material removal process of the single alumina particle with a diameter of 12.8 μm impacting the optical glass at the impact speed of 50 m/s and the impact angle of 90°. When the impacting alumina particle starts contacting the optical glass, the material removal is mainly realized by plastic mode, with no brittle crack forming on the surface or subsurface layers of the material, as shown in Figure 8a. However, as the impact process continues, transverse cracks appear around the impact craters and continue to expand, eventually stopping on the surface of the material and resulting in the generation of massive chips, as presented in Figure 8b. When the impact depth continues to increase, a radial crack perpendicular to the surface of the material can be clearly observed in the subsurface layer, which affects the subsequent use of the material, as shown in Figure 8c. Thus, the material removal exhibits significant brittleness removal characteristics when the single-particle alumina with a diameter of 12.8 μm impacts the optical glass at an impact velocity of 50 m/s and an impact angle of 90°. In detail, transverse cracks first occur, followed by radial cracks. The formation of transverse and radial cracks sharply reduces the quality of the material surface and subsurface layers. Based on the result of the SPH simulation, the brittle removal process of the single-particle alumina impacting the optical glass materials is summarized as follows:

1. The particles first cause plastic deformation of the material.
2. Transverse cracks form around the deformation zone and gradually expand to the surface of the material, which eventually leads to the generation of brittle chips, thereby causing material removal.
3. Radial cracks perpendicular to the surface of the material form in the subsurface layer and gradually expand to the deeper subsurface layer.

### 4.2. Effect of Impact Angle on Material Removal

**Figure 9:**
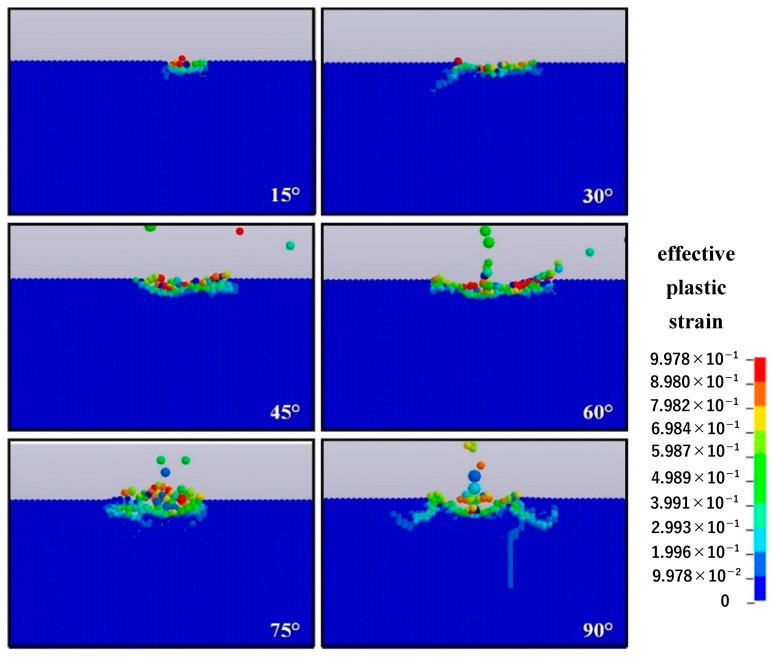
Surface morphology of impacted optical glass under different impact angles.

Figure 9 shows the surface morphology of the single-particle alumina with a diameter of 12.8 μm impacting the optical glass at an impact speed of 50 m/s and different impact angles (15°, 30°, 45°, 60°, 75°, and 90°). We can observe that the impact angle plays a crucial role in the material removal mode. When the impact angle is 15°, the contact area between the single-particle alumina and material surface is the smallest, which leads to a slight plastic deformation on the material surface. When the impact angle is 30°, microcracks are generated in the subsurface layer of the material. As the impact angle increases and settles between 45° and 75°, transverse cracks are produced due to the extrusion between the impact particles and material, thereby resulting in the generation of massive chips and leading to material removal. Nonetheless, when the impact angle is 45° or 60°, no radial cracks are observed in the subsurface layer of the material, whereas when the angle is 75°, shorter radial cracks are observed in the subsurface layer. The material removal process for the impact angle of 90° is described in Section 4.1. By comparing the surface morphology of the optical glass under different impact angles, we can observe that with the increase of the impact angle, transverse cracks that cause a large amount of material removal are first formed and no subsurface damage is caused. Conversely, when the impact angle is greater than 60°, radial cracks that affect the quality of the material are generated in the subsurface layer of the material.

**Figure 10:**
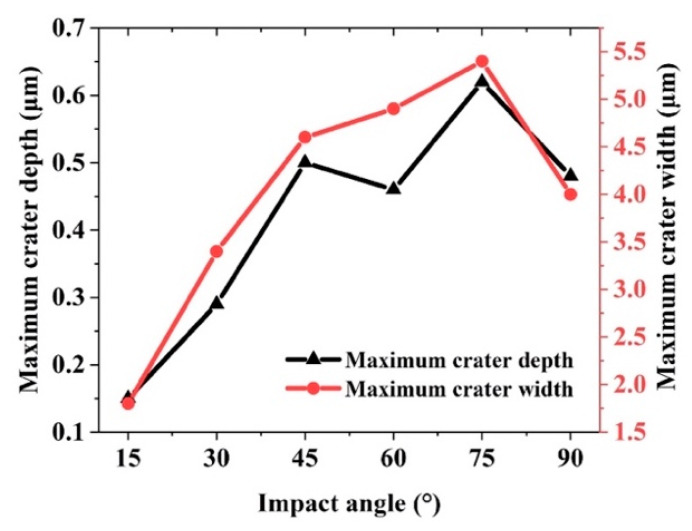
Maximum depth and width of craters under different impact angles.

Figure 10 quantitatively demonstrates the relationship between the maximum depth and maximum width of the craters on the material surface as the impact angle increases. Under the impact angles of 15°, 30°, 45°, 60°, 75°, and 90°, the corresponding maximum depths of the craters are 0.15, 0.29, 0.50, 0.46, 0.62, and 0.48 μm respectively, and the corresponding maximum widths of the craters are 1.8, 3.4, 4.6, 4.9, 5.4, and 4.0 μm, respectively. When the impact angle is 60°, the reason for the decrease in the impact depth may be the fact that most of the particle energy causes the generation of lateral cracks, resulting in a decrease in the impact depth for the impact angle of 60°. By analyzing the maximum depth and width of the craters, we find that under the same impact speed, when the impact angle is between 45° and 75°, the material removal rate is the highest and the increase of the removal rate does not cause subsurface damage.

### 4.3. Effect of Particle Diameter on Material Removal

**Figure 11:**
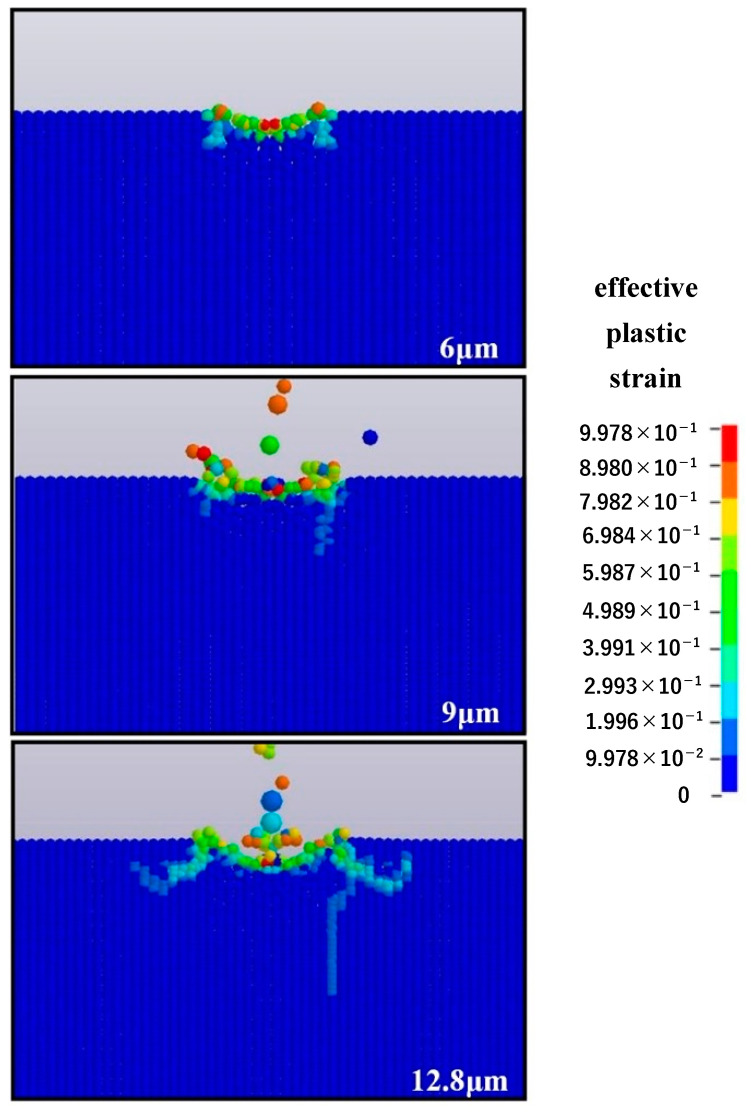
Surface morphology of impacted optical glass under different particle diameters.

Figure 11 displays the surface morphology of optical glass impacted by single-particle alumina with different particle diameters (6, 9, and 12.8 μm) at the impact speed of 50 m/s and the impact angle of 90°. The results show that the particle diameter greatly affects the material removal process. When the particle diameter is 6 μm, the material removal mainly depends on plastic deformation and no brittle crack is generated. However, when the particle diameter is 9 μm, short transverse cracks and radial cracks begin to form, indicating the occurrence of brittle removal, which is even more obvious when the diameter reaches 12.8 μm. Therefore, we can conclude that the increase of the particle diameter affects the material removal mode during the impact process. Furthermore, when the impact particle diameter is 6 μm, the material removal is mainly achieved by plastic mode.

**Figure 12:**
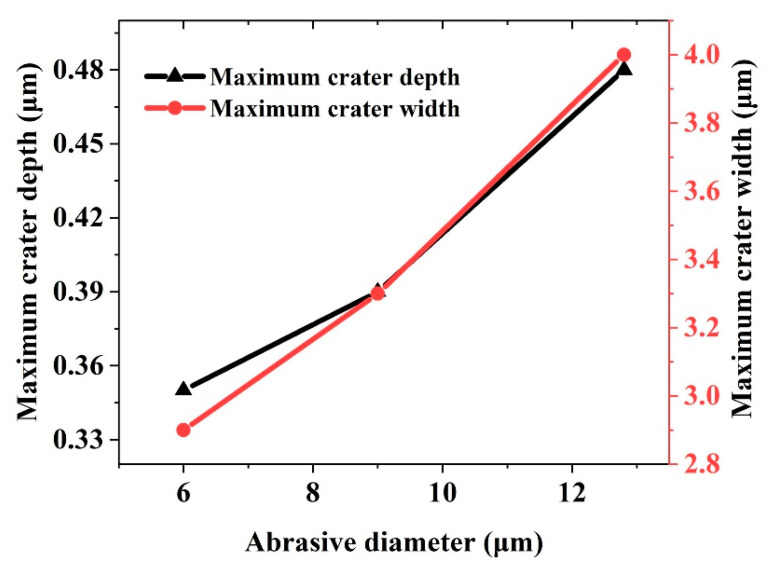
Maximum depth and width of craters under different particle diameters.

Figure 12 quantitatively illustrates the relationship between the maximum depth and maximum width of the craters on the material surface as the particle diameter increases. Under particle diameters of 6, 9, and 12.8 μm, the maximum depths of the craters are 0.35, 0.39, and 0.48 μm respectively, and the maximum widths are 2.9, 3.3, and 4.0 μm, respectively. By analyzing the maximum depth and width of the craters, we found that as the particle diameter increases, the material removal rate increases accordingly, but the increase leads to brittle failure.

### 4.4. Effect of Impact Parameters on Energy Loss of Particles

**Figure 13:**
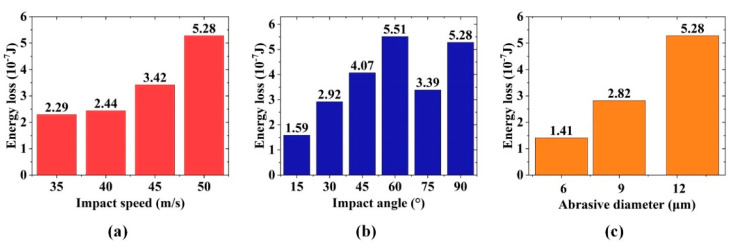
Energy loss changes under different impact parameters: (**a**) impact speed, (**b**) impact angle, and (**c**) particle diameter.

In order to understand the erosion mechanism of optical glass from the perspective of energy conversion, the energy loss was calculated by subtracting the rebound kinetic energy from the initial kinetic energy of particles. Figure 13 shows the effects of various impact angles and impact speeds on the energy loss of particles with a diameter of 12.8 μm. As the impact velocity increases, the energy loss also increases. When the impact speed is greater than 40 m/s, the loss begins to increase rapidly, which accounts for the condition that when the impact speed is greater than 40 m/s, brittle cracks occur on the material surface. Figure 13b shows that the energy loss increases when the impact angle increases from 15° to 60°. However, when the impact angle is greater than 60°, a decrease in the energy loss occurs, indicating that when the impact angle is approximately 60°, the material absorbs energy best. The sharp decrease in the energy loss for the impact angle of 75° may be influenced by the reduction in the contact area and the initiation of median cracks. This condition also explains, in terms of energy, that the material removal rate reaches the maximum when the impact angle is between 45° and 75°. Figure 13c shows that as the diameter of the impact particles increases, the energy loss also increases. The relationship between diameter and energy loss may be basically linear, reflecting that the increase in diameter can greatly improve the material removal rate. The results have guiding significance for the research on the polishing mechanism and process optimization.

## 5. Conclusions

In this study, the theoretical model of the single-alumina particle impacting the optical glass was established through SPH. The simulation was conducted under different impact speeds, impact angles, and particle diameters. The correctness of the simulation model was verified by single-particle impact experiments. The conclusions are as follows:

1. The increase of impact speed increases the width and depth of the craters, thereby enhancing the material removal rate. Moreover, when the impact speed is greater than 40 m/s, brittle removal occurs and microcracks are formed around the craters, thereby resulting in material breakage and affecting the surface quality of the material.
2. The dynamic evolution mechanism of crack initiation and propagation in the single-particle impact process of optical glass is clarified. The particles first cause plastic deformation of optical glass. As the impact process continues, transverse cracks are generated around the deformation zone and then gradually expand to the surface of the material. Meanwhile, radial cracks perpendicular to the surface of the material are produced in the subsurface layer and gradually expand to the deeper subsurface layer.
3. With the gradual increase of the impact angle, the transverse cracks that cause a large amount of material removal are first generated. When the impact angle is greater than 75°, radial cracks appear in the subsurface layer of the material. At the same impact speed, when the impact angle is between 45° and 75°, the material removal rate is the highest, and the increase in the material removal rate does not cause damage to the subsurface layer of the material.
4. The increase in particle diameter affects the material removal mode during impact. When the particle diameter is 6 μm, the material is removed by the plastic removal mode. As the particle diameter increases, the material removal rate increases accordingly but leads to brittle failure.
5. The energy loss increases with the increase of impact speed. When the impact speed is greater than 40 m/s, the energy loss begins to increase rapidly. The energy loss also increases when the impact angle increases from 15° to 60°. However, the energy loss begins to decrease when the angle is greater than 60°, which means that the material absorbs energy best at the impact angle of 60°. The increase in particle diameter also increases the kinetic energy loss correspondingly.

## Figures and Tables

**Table 1:** JH-2 parameters of optical glass [37].

Description	Symbol	Value
Density (kg/m^3^)	ρ	2530
Shear modulus (Gpa)	G	30.4
Intact normalized strength coefficient	A	0.93
Intact strength exponent	N	0.77
Fractured normalized strength coefficient	B	0.088
Fractured strength exponent	M	0.35
Strain rate coefficient	C	0.003
Net compressive stress at HEL (GPa)	HEL	5.95
Pressure component at HEL	$p_{HEL}$	2.92
Maximum tensile strength (GPa)	T	0.15
Reference strain rate	$\varepsilon_{0}$	1
Maximum fracture strength	SFMAX	0.5
Damage coefficient	D1	0.053
Damage exponent	D2	0.85
Bulk modulus (GPa)	K1	45.4
Pressure coefficient (GPa)	K2	−138
Pressure coefficient (GPa)	K3	290

**Table 2:** Simulation parameters of a single particle impacting the optical glass.

Impact Parameters	Value
Impact speed (m/s)	30, 35, 40, 45, 50, 55
Impact angle (°)	30, 45, 60, 75, 90
Abrasive diameter (μm)	6, 9, 12.8
